# Late 21st‐Century Climate and Land Use Driven Loss of Plant Diversity in African Mountains

**DOI:** 10.1111/gcb.70492

**Published:** 2025-09-22

**Authors:** João de Deus Vidal Junior, Alexandre Antonelli, Clinton Carbutt, Vincent Ralph Clark, Tobias Fremout, Christopher Chapano, Inês Chelene, David Chuba, Tadesse Woldemariam Gole, Clayton Langa, Benoit Loeuille, Ermias Lulekal Molla, Timothy Richard Pearce, Andrew J. Plumptre, Feyera Senbeta, Carolina Tovar, Joseph Douglas Mandla White, Christine Brigitte Schmitt

**Affiliations:** ^1^ Geography Section University of Passau Passau Germany; ^2^ Afromontane Research Unit & Department of Plant Sciences University of the Free State Phuthaditjhaba South Africa; ^3^ Royal Botanic Gardens, Kew Richmond UK; ^4^ Gothenburg Global Biodiversity Centre, Department of Biological and Environmental Sciences University of Gothenburg Gothenburg Sweden; ^5^ Department of Biology University of Oxford Oxford UK; ^6^ Chinese Academy of Sciences Wuhan Botanical Garden Wuhan China; ^7^ School of Agriculture and Science University of KwaZulu‐Natal Scottsville South Africa; ^8^ Scientific Services, Ezemvelo KZN Wildlife Cascades South Africa; ^9^ Alliance Bioversity International—CIAT Lima Peru; ^10^ National Herbarium of Zimbabwe Harare Zimbabwe; ^11^ Herbário LMA Do Instituto de Investigação Agrária de Moçambique Maputo Mozambique; ^12^ Department of Biosciences and Biotechnology University of Zambia Lusaka Zambia; ^13^ Environment and Coffee Forest Forum (ECFF) Addis Ababa Ethiopia; ^14^ Department of Plant Biology and Biodiversity Management Addis Ababa University Addis Ababa Ethiopia; ^15^ Key Biodiversity Area Secretariat, c/o BirdLife International Cambridge UK; ^16^ Department of Biological Sciences Botswana University of Agriculture and Natural Resources Gaborone Botswana

**Keywords:** climate change, conservation biology, distribution modelling, habitat loss, mountain biodiversity

## Abstract

With the 1.5°C–5°C increase in global temperature projected for this century, many plant species are expected to shift their distribution ranges to track their environmental requirements. Across several mountain regions, responses to climate change like upslope shifts may result in accelerated rates of species turnover, species richness increases in upper montane belts, and amplified habitat losses. Yet, evidence of how such processes may influence plant diversity in Africa is still scarce. Here, using a species distribution modeling approach, we quantify and map how different scenarios of climatic and land‐use changes may affect plant species ranges in African mountains. Using individually tuned models and dispersal buffers, we compared distribution losses and potential expansion through dispersal across 607 vascular plant species under three shared socioeconomic pathways for the end of the century. Our projections indicate that keeping warming under 2°C until 2100 under a sustainability scenario (SSP1.26), almost half (49.3%) of the species would experience a contraction in suitable areas, compared to 71%–75.6% in case these targets are not met (SSP3.70 and SSP5.85). Among these losses, mean contractions between 19% and 50.4% are predicted depending on the scenario. We project rates of upslope shifts that may be up to three times higher than the global calculated average. Contractions will be higher for species occurring at upper elevations, and trees and shrubs will show lower declines. Our findings align with previously reported trends of upslope shifts of species distributions but suggest that accelerated rates of change may limit the capacity of some species to track their niche based solely on their natural dispersal capacity. This implies that further efforts to improve habitat connectivity, restoration, and assisted migration may be necessary.

## Graphical abstract

By the end of this century, many African mountain plants may lose large parts of their suitable habitats as the climate warms and land use changes. Even under a sustainable pathway limiting warming to under 2°C, nearly half of the 607 studied species are projected to contract their ranges, with losses even greater under higher emission scenarios. Species are expected to shift upslope at unusually fast rates, but many may not keep pace naturally, highlighting the need for conservation strategies like improving habitat connectivity, restoration, and assisted migration to safeguard mountain plant diversity.

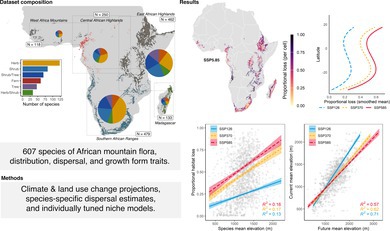

## 1 Introduction

A primary goal of the Kunming‐Montreal Global Biodiversity Framework for 2050 is to halt anthropogenic extinctions (CBD 2022). Mountains are regions where achieving this objective is critical due to their outstanding diversity—generated over millions of years of orogenic evolution (Antonelli et al. 2018; Hoorn et al. 2018; Rahbek et al. 2019)—and the accelerated rates of climate warming observed at high elevations over decades (Adler et al. 2022; Pepin et al. 2015). Globally, climate change and land‐use change, along with direct exploitation and invasive species, are ranked among the most impactful current drivers of change in terrestrial biodiversity (Díaz et al. 2019).

The capacity of a species to avoid extinction depends, among other factors, on its physiological tolerance, genetic adaptability, and ability to disperse and migrate to suitable habitats under novel climatic conditions. Range responses to climatic changes like migration are deeply influenced by species' current geographic distribution and life history traits (Compagnoni et al. 2021; Vincent et al. 2020). Many plants show delayed migration paces compared to other groups due to relatively long generation times and limited dispersal distances, indicating that they may be unable to track their suitable environments under the accelerated ongoing warming (Corlett and Westcott 2013). In the absence of adaptation (i.e., evolutionary change in environmental tolerances) or acclimation (i.e., physiological change in environmental tolerances of individuals), this mismatch results in fitness declines and, ultimately, an increased risk of local extinction (Anderson and Wadgymar 2020). Due to the spatial distribution of environmental conditions across the elevation gradient, this may be particularly concerning for species restricted to mountains (Dullinger et al. 2012). Alternatively, the presence of conservative traits adapted to harsh environmental conditions in high‐elevation floras has been suggested to enhance their resilience to climate change (Körner and Hiltbrunner 2021). To understand how different elements of the African mountain flora may resist or perish under such pressures, it is necessary to analyze simultaneously their habitat availability and dispersal capacities, which may improve conservation strategies tailored to mitigate biodiversity loss in the region.

The Sub‐Saharan African mountains encompass at least five biodiversity hotspots (Myers et al. 2000), which include mountain ranges like those in the Greater Cape Floristic Region, the Maloti‐Drakensberg/Eastern Great Escarpment, Mount Cameroon, and the Eastern Arc Mountains (Sosef et al. 2017; Trisos et al. 2022). Spanning from 34.6° S to 37.3° N and occupying approximately 1,863,000 km^2^ of land between 500 and 5895 m above sea level, these mountains include a highly diverse combination of montane and alpine floras (Linder 2014; White 1978). Besides being biologically unique, they also directly provide water, energy, and livelihoods to more than 227.8 million people living in these mountain regions (Adler et al. 2022). As ecosystem services rely directly on biodiversity, declines in species numbers and regional extinctions may disrupt their delivery (Midgley 2012). Despite that, the geographical distribution of plant diversity in Africa is poorly documented compared to the rest of the tropics (Ondo et al. 2024). On other continents, long records of surveys for mountain floras depict recent species composition shifts linked to warming temperatures (Iseli et al. 2023; Morueta‐Holme et al. 2015; Steinbauer et al. 2018), with extinctions projected and reported for mountain species across taxonomic groups (Kidane et al. 2019; Parmesan 2006). Simultaneously, invasive species are rapidly colonizing upper elevations (Iseli et al. 2023; Pauchard et al. 2009), imposing additional pressures on the persistence of local species. While similar long‐term studies are still scarce for African mountains, existing models indicate that, by 2085, temperature and precipitation changes may cause range shifts for 81 to 97% of the plants on the continent (McClean et al. 2005).

Mountain plants are amongst the most vulnerable groups to climate‐driven extinction in Africa (Trisos et al. 2022), due to their sessility and limited niche breadth (Dirnböck et al. 2011), and accelerated warming in African mountains compared to global rates (+0.3°C/decade between 1991 and 2022) (*State of the Climate in Africa 2022*, 2023). Despite its urgency, forecasting African mountain species' responses to climate change is particularly challenging due to the limited availability of digitized occurrence records (Clark et al. 2011; Ondo et al. 2024). Species distribution models (SDMs) have been widely used to predict climate change impacts on species distributions (Ivory et al. 2016; Kidane et al. 2022). This approach is based on the idea that climatic niches can be estimated from distribution data via statistical modeling, which can then be projected to future climates. Surprisingly, dispersal is often overlooked in such projections (Barve et al. 2011), although species are only able to colonize areas within their accessible ranges (Sánchez‐Fernández et al. 2016). Similarly, land use also impacts the proportion of suitable habitat that may be accessible and occupied by species. Including these constraints to model projections significantly enhances the ecological relevance of SDMs (Barve et al. 2011). However, the few studies that attempted to predict the impact of climate change on the vegetation of African mountains are limited to a few species or regions (Bentley et al. 2019; Thuiller et al. 2006), and did not include dispersal estimates or land use change scenarios. We aim to fill this gap by comparing the responses of multiple species across African mountain regions using a combination of species distribution models, land use change, and dispersal estimates to improve our understanding of climate change impacts on the range dynamics of plants occurring in African mountains.

To achieve this, we modelled and compared the distribution responses of 607 species spanning 112 mountain ranges in 41 Sub‐Saharan African countries, including species with a wide range of mean elevations (340–2218 m). We focused our comparisons on native African species with the highest share of their distributions occurring in mountains (as defined by the Global Mountain Biodiversity Assessment version 2.0; Snethlage et al. 2022). We projected each of the species distributions to end‐of‐the‐century (2100) combining climatic conditions and land use classes under three climate change scenarios, namely SSP1.26 (Sustainability scenario, with anthropogenic radiative forcing of 2.6 W/m^2^ by 2100), SSP3.70 (Regional Rivalry scenario, 7 W/m^2^ by 2100), and SSP5.85 (Fossil Fueled Development scenario, 8.5 W/m^2^ by 2100), limiting potential expansions according to each species dispersal capacity and habitat availability with land use change.

Our models indicate that under a sustainable land use scenario, keeping emissions compatible with the 2°C target by 2100 (SSP1.26) would mitigate distribution losses in comparison with higher warming and other land use scenarios. Overall, upslope shifts are predicted to be the most common response, while no clear pattern of latitudinal shift could be identified. In particular, species associated with higher elevation ranges are projected to be exposed to higher distribution losses and extinction risks but have the potential to compensate for such losses via latitudinal and elevational expansion through dispersal. Such a scenario, however, will depend on habitat connectivity and ecological interactions, which are also expected to be impacted by climate change. Our projections show a considerable variety of responses across life forms, elevation ranges, and regional species richness, providing a first continent‐wide synthesis of African plant species to climate and land use change in mountains.

## 2 Materials and Methods

### 2.1 Occurrence Records Compilation and Data Cleaning

We compiled occurrence records for vascular plants found in Africa using information from regional herbaria (GRA, SRGH, LMA, UZL, UCBG, ETH), literature sources, unpublished surveys by The Wildlife Conservation Society (WCS) in the Albertine Rift, and online repositories (GBIF.org 2023). We removed occurrences with only genus‐level identification, assigned valid names to synonymized records, obtained family names for each species, and standardized names using the TNRS package version 0.3.1 (Maitner et al. 2023). We collapsed all taxonomic classifications below the species level and removed hybrids from the dataset. We used this curated species list in the following steps to obtain range information for each species. We overlaid these records with the native range distributions of each species from the Plants of the World Online website (https://powo.science.kew.org/) using the function “search_powo” from the “kewr” package version 0.6.1 (Walker 2022) and the World Geographical Scheme for Recording Plant Distributions (WGSRPD, https://github.com/tdwg) framework. Using the R package “terra” version 1.6–17 (Hijmans, Bivand, et al. 2022), we applied these ranges to classify and remove species considered non‐native to Sub‐Saharan Africa and to flag and remove occurrences outside the native range of each species. We also applied a temporal filter by restricting the occurrences to those collected after 1981 to match the time frame of the environmental datasets. We removed and filtered records with invalid coordinate values, records matching country capitals, centroids, coordinates with zero or equal values for latitude and longitude, records matching institutions, and records corresponding to water bodies using the R package “CoordinateCleaner” version 2.0–20 (Zizka et al. 2019). To minimize the impact of spatially biased presence coordinates on model calibration, we removed records falling within the same 2.5 arcmin grid cells.

### 2.2 Identification of Mountain‐Associated Species Suitable for Modelling

We analyzed a data set including 1,964,910 distribution points for all plant species recorded in Sub‐Saharan Africa. To identify mountain species to be included in our study, we first identified species with distributions primarily within mountain ranges and having sufficient records as described below. We limited the geographic extent of the environmental predictors to Sub‐Saharan Africa and Madagascar, using the Afrotropical realm shapefile as a mask (Olson et al. 2001).

To calculate a threshold for the selection of mountain‐associated species, we first calculated the overlap extent of each species buffer located within a region classified as a mountain by the global mountains shapefile (narrow delimitation) version 2.0 (Snethlage et al. 2022) developed by the Global Mountain Biodiversity Assessment (GMBA, http://www.mountainbiodiversity.org/). The GMBA 2.0 dataset adopts a morphometric approach based on digital elevation models and provides a globally standardized delineation for mountain regions. We then generated a polygon around the records of each species by creating a 1 km buffer and dissolving the features. We cropped the dissolved polygons to the mountain shapefile and applied the “expanse” function available on the terra package to obtain the polygon areas in kilometers. We divided the cropped area by the total area and then summarized the distribution of all proportional mountain areas for all the species in the dataset.

After applying multiple data‐cleaning steps to remove non‐native species, incomplete entries, geographic outliers, and records beyond the temporal range of the study and the natural ranges of each species, we obtained a dataset comprising 419,055 occurrences. From this dataset, we retrieved the distributions of 27,789 species occurring within mountains, of which 7376 were restricted to mountains. We retained only species with at least 14 non‐overlapping (i.e., occurring in different grid cells) records to approximate the minimum requirement for modeling distributions of narrow‐ranged species demonstrated previously for African species (van Proosdij et al. 2016), from which we selected only those species with more than 38% of their distribution within mountains, corresponding to the 75th percentile of the proportion of distribution in mountains of all species native to Sub‐Sahara Africa (Table 1).

**TABLE 1:** Dataset overview.

Metrics	SSP1.26	SSP3.70	SSP5.85
Species with net range loss (%)	49 (*n* = 299)	71 (*n* = 431)	76 (*n* = 459)
Species with net range gain (%)	51 (*n* = 308)	29 (*n* = 176)	24 (*n* = 148)
Species with upward elevational shift (%)	75 (*n* = 456)	84 (*n* = 509)	85 (*n* = 514)
Species with downward elevational shift (%)	25 (*n* = 151)	16 (*n* = 98)	15 (*n* = 93)
Species increasing southern limit (%)	45 (*n* = 270)	45 (*n* = 271)	44 (*n* = 268)
Species increasing northern limit (%)	31 (*n* = 189)	45 (*n* = 271)	49 (*n* = 297)
Mean proportional range loss (%)	–25	−47	−55
Mean proportional range gain (%)	82	68	62

*Note:* Summary of modelled changes for 607 species of plants occurring across African mountains under different climate and land use change scenarios (shared socioeconomic pathways SSP1.26, SSP3.70, SSP5.85) for 2100.

After filtering species that had insufficient records to produce reliable suitability models, we selected 607 species, representing 396 genera and 115 families, for which we compiled information on dispersal syndromes and life forms. The complete dataset of geographic records and dispersal information is available as Data S1.

### 2.3 Maximum Dispersal Buffer Calculation

We used the custom function “dispeRsal” (Tamme et al. 2014), which applies a linear regression using traits and species taxonomy data to estimate the maximum dispersal distances per generation for each species. We classified life forms for each species in the dataset from Flora Zambesiaca (https://plants.jstor.org/collection/FLOZAM), Flora of Tropical East Africa (https://plants.jstor.org/collection/FTEA), and Flora of West Tropical Africa (https://plants.jstor.org/collection/FLOWRS). Life forms were also obtained from the Kew Plants of the World Online website (https://powo.science.kew.org/) for species not included in these publications or those missing this information. We classified each species to match the algorithm input according to the following categories: shrubs (including shrubs, climbing shrubs, and subshrubs), trees (including trees and small trees), herbs (including perennial, annual, epiphyte, hemiparasitic epiphyte, and tuberous geophyte). Dispersal syndromes were obtained for each species or, when unavailable, at the genus level using the descriptions in these Floras or literature sources. The full references are available as Data S1. To match the dispersal information of each species with the inputs of the “dispeRsal” function, we classified each species into the following categories, in ascending order of maximum dispersal distance: wind (not‐adapted), ballistic, ant, wind (adapted), or animal. When species fit multiple categories, we considered the category with the highest dispersal distance. Since this package does not have a specific category for modeling the dispersal of ferns, we modeled them using the same parameters as herbaceous plants with a wind‐specialized dispersal mechanism. We also obtained the IUCN Red List classification for each species using the R package “rredlist” version 0.7.1 (Gearty and Chamberlain 2022).

To produce a polygon simulating the total maximum dispersal distance of each species into the future (2100, ca. 70 years from present), we scaled the calculated dispersal distances estimated in the previous step using reproductive maturity ages to account for the cumulative range added across multiple colonization events. We obtained minimum juvenile periods (in years) for 249 species in genera available in the literature (Moles et al. 2004). When information was not at the genus level, we calculated average values based on life forms. The distances multiplied by the maximum generation number values were then used as the distance parameter in the “buffer” function of the terra package, which was applied over the present scenario models to produce masks for the future environmental layers in the projection step for each future model. This step produced individually cropped future layers, which were scaled according to the maximum dispersal ranges of each species. Resulting dispersal buffers were then cropped to restrict trans‐oceanic migration, with potential dispersal buffers being discarded for islands not present in the species' current distribution according to POWO.

The three main families represented in our dataset were Fabaceae, Asteraceae, and Rubiaceae (Data S1). The most frequent dispersal syndrome classes (following the parameters available on the dispersal function) were animal dispersal (including epizoochory and endozoochory), and the least frequent was ant dispersal. For life forms, herbs were most common (275 species classified as herb and 50 classified as herb or shrub), followed by shrubs (137) and trees (66), while 90 species were classified as shrub or tree.

### 2.4 Climate, Soil, and Topographic Datasets

We downloaded a set of 46 climatic layers available for present and future climatic scenarios at a 30‐arc‐second resolution from CHELSA version 2.1 (https://chelsa‐climate.org/) (Karger et al. 2018), which has been reported to produce better results than other climatic products for modeling studies in mountain regions (Bobrowski et al. 2021). To reduce model complexity, we selected a subset of nine temperature and four precipitation variables: mean annual air temperature (bio1), mean diurnal air temperature range (bio2), isothermality (bio3), temperature seasonality (bio4), mean daily maximum air temperature of the warmest month (bio5), mean daily maximum air temperature of the coldest month (bio6), annual range of air temperature (bio7), mean daily mean air temperatures of the warmest quarter (bio10), mean daily mean air temperatures of the coldest quarter (bio11), annual precipitation (bio12), precipitation amount of the wettest month (bio13), precipitation amount of the driest month (bio14), and precipitation seasonality (bio15). We also included nine 30‐cm‐depth soil predictors, namely organic carbon density (ocd), soil organic content (soc), bulk density (bdod), cation exchange capacity (cec), clay content (clay), nitrogen content (nitrogen), pH in H_2_O (phh2o), sand content (sand), silt content (silt), downloaded from the SoilGrids 2.0 dataset (Poggio et al. 2021). We used the SRTM 30‐m digital elevation model to interpret the model results within the context of each mountain, which was aggregated to 2.5 arc minutes in the modeling step.

We projected individual species distribution models into the three SSP scenarios available in CHELSA, namely SSP1.26, SSP3.70, and SSP5.85, using five different Global Circulation Models (GCMs) projections to maximize model diversity and reduce model selection bias (Sanderson et al. 2015). These GCMs are Geophysical Fluid Dynamics Laboratory—Earth System Model version 4.0 (GFDL‐ESM4), UK Met Office Earth System Model version 1.0 (UKESM1‐0‐LL), Institut Pierre Simon Laplace—Climate Model Version 6 (IPSL‐CM6‐LR), Meteorological Research Institute—Earth System Model version 2 (MRI‐ESM2), and the Max Planck Institute Earth System Model (MPI‐ESM1‐2). We adopted a majority consensus criterion, only considering areas identified as presences in at least three of the five GCMs in the final map.

To assess human impacts and map vulnerability, we also applied global land‐use change projections based on the Global Change Analysis Model (GCAM) (M. Chen et al. 2020) as a mask to project future distributions to account for future habitat availability. We used land‐use change model means (i.e., the mean values for five global climate models available in the dataset, namely GFDL, HADGEM, IPSL, MIROC, and NORESM) for each scenario, using land‐use classes in SSP2.45 (“middle of the road” 4.5 W m^−2^ of radiative forcing by 2100, assumed to be the most probable scenario; Williams et al. 2024) for 2020 as a baseline comparison. We compared the highest percentage value per cell between all land cover classes for each SSP to identify the dominant type in each 0.05° cell. We masked the future projections of each species, removing cells dominated by land‐cover categories we interpreted as unlikely to be available for species to occupy: corn, wheat, soybean, cotton, rice, sugar crop, other crops, bioenergy crop, and urban.

### 2.5 Species Distribution Models Calibration, Projection, and Evaluation

We generated tuned species distribution models using the Maximum Entropy (MaxEnt) algorithm available on the R package “ENMeval” version 2.0.4 (Kass et al. 2021). To calibrate the models, we sampled 10,000 random background points within the current distribution range according to POWO, using the WGSRPD geographic classification. To evaluate the models, we used a five‐fold cross‐validation using 250‐km wide blocks to divide the data into training and testing data, using the package blockCV version 3.1 to 3 (Valavi et al. 2022). We then used the “evaluate” function from the “dismo” package version 1.3 to 9 (Hijmans, Phillips, et al. 2022) to quantify the goodness‐of‐fit of the models using the Area Under the receiver operating characteristic Curve (AUC) and sensitivity/specificity metrics. Variable importance values for the highest performing model (in terms of AUC) were calculated with ENMevaluate() using variable permutation importance and percent contribution.

Models were considered acceptable when AUC values were higher than or equal to 0.70, with lower performing models being discarded from further analysis steps. To map losses and gains in suitability for each species, we converted models into presence‐absence maps using the maximum specificity/sensitivity as a threshold (Liu et al. 2005). Models resulting from the candidate species dataset showed average AUC values ranging from 0.58 to 0.99. The full model evaluation statistics are available as Data S1. After removing 11 poor‐performing species models (i.e., below the AUC threshold of 0.7), 607 species were kept for the downstream analysis.

Using the distribution models generated in the first step, we calculated present and projected species richness using the R package “terra.” We overlapped all binary (presence‐absence) maps for each climatic scenario and calculated the difference between each future stack and the present scenario. For the present models, we extracted latitude and elevation values for each cell identified as a presence and used the values to calculate mean, maximum, and minimum ranges for each species. We performed all geographic analyses under a spatial resolution of 2.5 arc minutes and used Mollweide projection to minimize area distortion. We opted to present the visualization of the changes in richness (Figure 3b–d) clipped to above 5% of the total richness (i.e., only showing areas with more than 30 species) to minimize the bias induced by individual species responses on the identification of regional changes in the target mountains.

## 3 Results

### 3.1 Species Range Responses

An overview of the taxonomy, dispersal syndrome, and life form of the modelled species is available as Data S1. We compared the modelled distribution of each species based on (I) losses, (II) gains, and (III) net change (gains minus losses) (Table 2). Additionally, for the present and future distributions, we also compared the mean elevation (IV) and latitudinal (V) ranges. The complete summary of individual species distribution losses, gains, elevation, and latitudinal shifts is listed as Data S1.

**TABLE 2:** Response summary.

Mountain region	Species included (*n* = 607)^a^	Total species (*n* = 27,789)^a^	Endemics included (*n* = 167)	Total endemics (*n* = 22,632)
Southern African Ranges	479 (33.2%)	12,425 (39.8%)	110 (65.9%)	10,450 (46.2%)
East African Highlands	462 (32.0%)	7040 (22.6%)	33 (19.8%)	4215 (18.6%)
Madagascar	133 (9.2%)	5699 (18.3%)	22 (13.2%)	5048 (22.3%)
Central African Highlands	250 (17.3%)	4129 (13.2%)	2 (1.2%)	2237 (9.9%)
West Africa Mountains	118 (8.2%)	1908 (6.1%)	0 (0.0%)	682 (3.0%)

*Note:* Summary of the geographic distribution and endemism of the 607 species of plants modelled, and the richness and endemism of native plant species in the complete dataset (419,055 records). *Species Included*: The number of modeled species that occur in each area. *Total Species*: The number of species in the initial dataset (before filtering and modeling) occurring in each region. *Endemics included*: The number of modeled species restricted to each mountain region. *Total endemics*: The number of species in the initial dataset restricted to each mountain region. Percentages represent proportion in relation to the column total.

^a^ Species can occur across several mountain regions.

**FIGURE 1:**
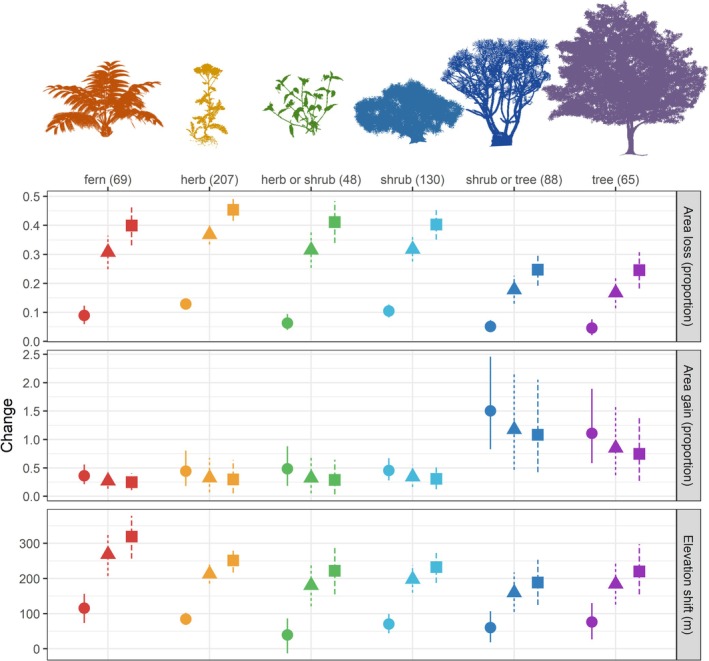
Projected distribution loss, gain, and elevation shift by growth forms across the 607 modelled species across different SSPs. The circles, triangles, and squares represent the mean values per scenario (● SSP1.26, ▲SSP3.70, ■ SSP5.85), and the vertical dotted lines represent the 95% confidence interval calculated with bootstrap (10,000 repetitions).

Overall, trees and shrubs showed lower losses and elevation shifts than other life forms (T‐statistic for mean net change: 2.35, T‐statistic for elevation shift: −2.32, degrees of freedom: 605, *p* < 0.05, Figure 1). Under SSP5.85, 459 species were predicted to have a negative net change in distribution as, for example, losses exceeding 90% for *Ajuga ophrydis* (animal dispersal herb, Lamiaceae), *Cerastium capense* (wind‐adapted dispersal herb, Caryophyllaceae), *Cussonia paniculata* (animal dispersal tree, Araliaceae), *Cynorkis debilis* (wind‐adapted dispersal geophyte, Orchidaceae), *Melica racemosa* (animal dispersal grass, Poaceae), and *Tina striata* (animal dispersal tree, Sapindaceae). Under SSP3.70, 431 species were predicted to have a negative net change, with *C. paniculata* and *T. striata* still showing losses higher than 90%. These negative net changes were considerably lower in SSP1.26, with only 299 species predicted to lose suitable area, with the highest projected loss being 63% for *C. paniculata* . Considering all species and all scenarios, average distribution losses were −25%, −47%, and −55% and average net changes were +57%, +20%, and +7% for SSP1.26, SSP3.70, and SSP5.85, respectively. When considering only species showing negative net changes, the mean proportional losses ranged from −19%, −42%, and −50% for SSP1.26, SSP3.70, and SSP5.85, respectively.

Positive net changes were less frequent, primarily projected for shrubs and trees under lower emission scenarios: 148, 176, and 308 species for SSP5.85, SSP3.70, and SSP1.26, respectively. *Maytenus peduncularis* (animal dispersal shrub or tree, Celastraceae) exhibited the highest average net distribution changes, with a gain of 33% across all scenarios. Similarly, *Helichrysum appendiculatum* (wind‐adapted dispersal herb, Asteraceae) and *Burchellia bubalina* (animal dispersal, Rubiaceae) also showed gains across all SSPs (28% and 19% respectively in SSP1.26, 29% and 19% respectively in SSP3.70, and 28% and 19% respectively in SSP5.85). *Peddiea africana* (animal dispersal tree, Thymelaeaceae) also showed consistent gains, but only under the moderate and high emission scenarios (13% in SSP3.70 and 13% in SSP5.85).

**FIGURE 2:**
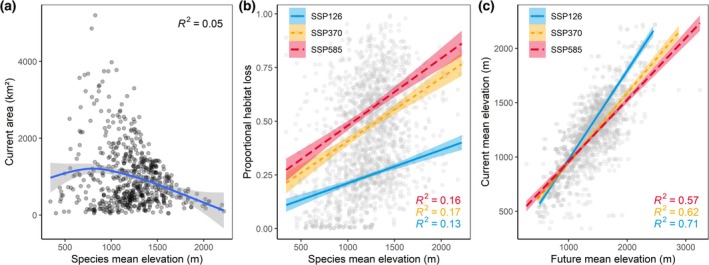
The relationship between range sizes, mean elevation ranges, and distribution loss for 607 mountain species. (a) Relationship between current area extent modeled with environmental variables and species mean elevation range (*R* = −0.29, *p* < 0.05). (b) Relationship between proportional habitat loss and species mean elevation range under three different SSPs (*R* = 0.36, *R* = 0.47, *R* = 0.51, *p* < 0.05). (c) Relationship between current and future species mean elevation ranges SSPs (*R* = 0.82, *R* = 0.79, *R* = 0.77, *p* < 0.05).

Mean elevation ranges for 456, 509, and 514 species were predicted to increase depending on the SSP. The mean elevation increases were 77, 203, and 240 m for SSP1.26, SSP3.70, and SSP5.85, respectively. Comparing multiple species trends with elevation, we observed that species with higher mean elevation ranges occupied, in general, smaller geographic areas (Figure 2a) and had higher distribution loss projections (Figure 2b). The magnitude of upslope shifts was also increased under higher warming scenarios (Figure 2c).

### 3.2 Regional Variations in Species Richness

**FIGURE 3:**
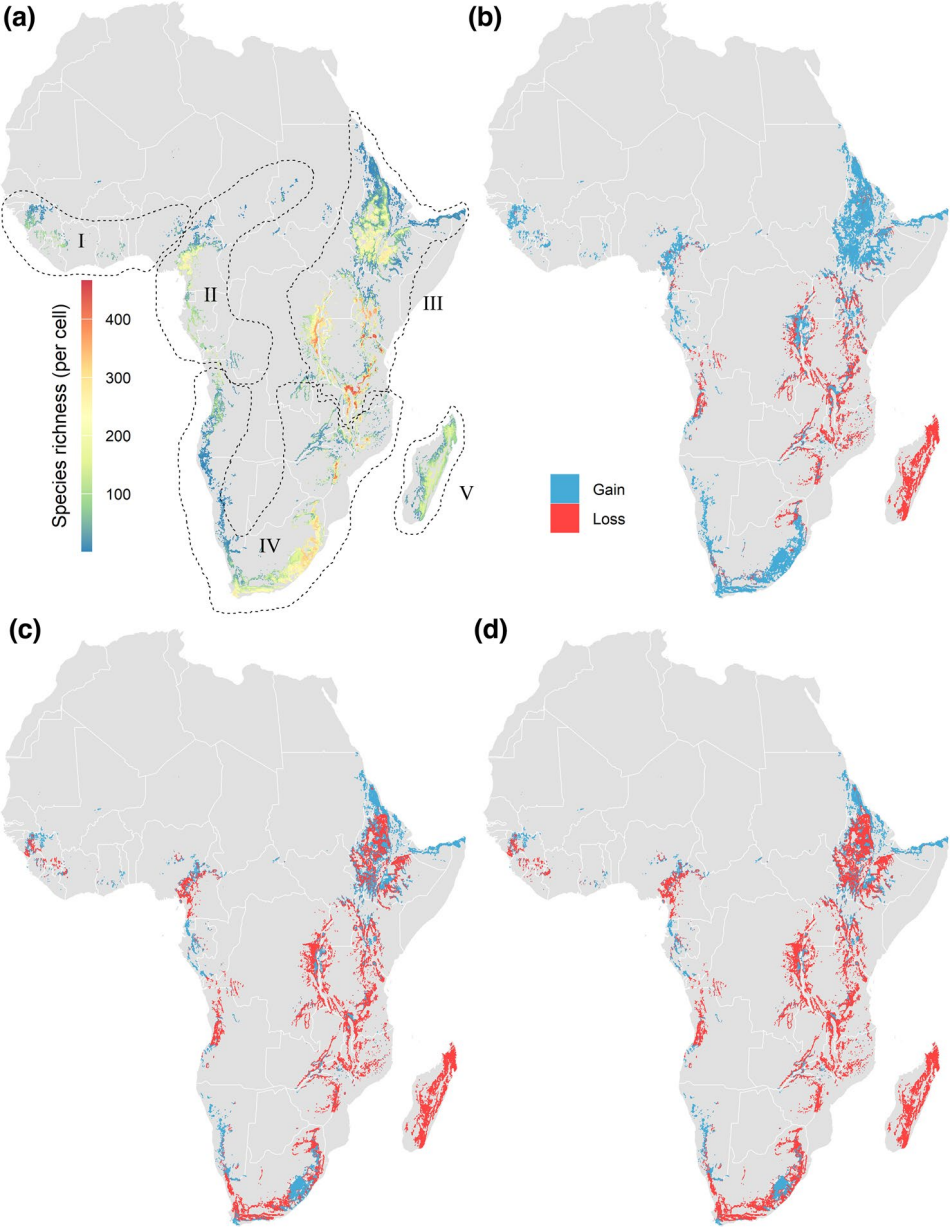
Current species richness and projected change in species richness per cell (2.5 arc‐min, ca. 5 × 5 km) for the 607 species analyzed, considering range expansions and contractions, according to the maximum dispersal capacity of each species in each of the compared SSPs: (a) The current number of species modeled for each cell, and change projected in species richness for (b) SSP1.26, (c) SSP3.70, and (d) SSP5.85. The regions delimited by the dashed lines follow level 2 of the GMBA classification, which we adopted for the geographic discussion of the results: (I) West Africa Mountains, (II) Central African Highlands, (III) East African Highlands, (IV) Southern African Ranges, and (V) Madagascar. Items 3b, 3c, and 3d were clipped to above 10% of the total richness (i.e., only showing areas with more than 40 species) to minimize biases induced by individual species responses in areas of lower richness. Map lines delineate study areas and do not necessarily depict accepted national boundaries.

Regional changes in mountain‐associated species richness for each scenario show uneven distributions of species richness gains and losses (Figure 3). Areas with the greatest declines in per‐cell species richness across all the scenarios were identified in the Eastern Arc, mainly in Kenya, the Democratic Republic of Congo, and Uganda, and the northwestern part of the Southern Ranges, mainly in Zambia, Malawi, Zimbabwe, and Mozambique. Under the SSP3.70 and SSP5.85 scenarios, areas with declines in species richness were also identified in Ethiopia, South Sudan, Angola, Cameroon, and South Africa. Comparatively, the West African Mountain region, represented in our dataset by species occurring in mountains like the Guinea Highlands, Volta Highlands, and parts of the Nigerian Highlands, showed increases in species richness projected in all scenarios.

**FIGURE 4:**
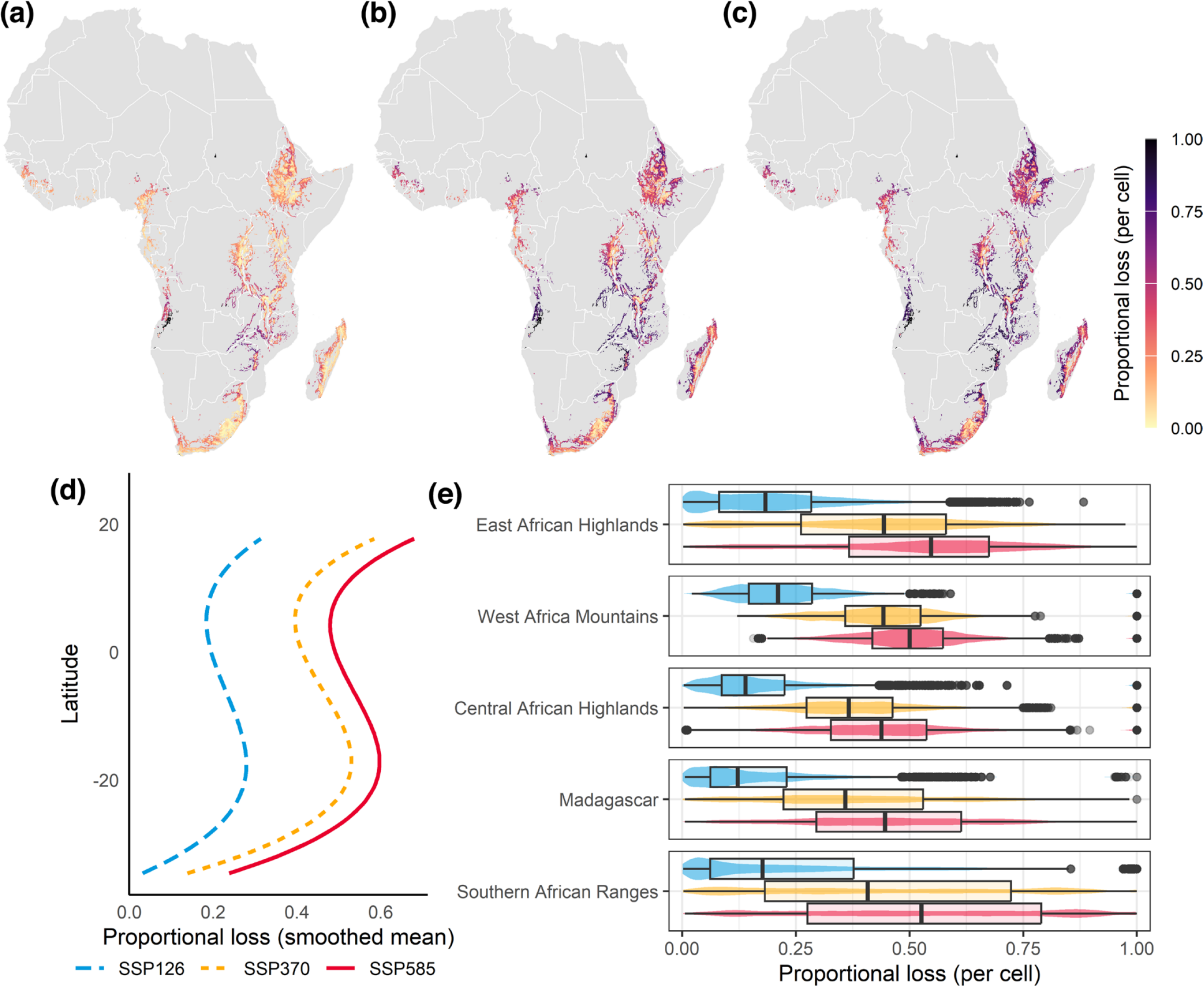
Distribution losses projected for 607 mountain‐associated species under three different SSPs for each grid cell within the study region (excluding range expansions). (a) SSP1.26, (b) SSP3.70, and (c) SSP5.85; (d) smoothed conditional mean values per latitude; and (e) regional boxplots showing minimum, lower hinge, median, upper hinge, and maximum loss values per 5 km cell. The values represent the projected number of species showing range loss in each cell in a given SSP divided by the number of species indicated as present in that cell under current climatic conditions. Map lines delineate study areas and do not necessarily depict accepted national boundaries.

When considering only areas currently classified as suitable that may become unsuitable (i.e., excluding range expansions), the proportional loss per cell changed substantially (Figure 4a–c). Losses were consistent with the prediction of higher declines for high emission scenarios (SSP5.85) compared to moderate and low scenarios (SSP3.70 and SSP1.26). Under such conditions, higher richness losses were projected for Madagascar, the East African Highlands, and the Southern African Ranges under the high and moderate emission scenarios (SSP5.85 and SSP3.70) (Figure 4e). Under the sustainability scenario (SSP1.26), Madagascar, the North African Highlands, and the East African Highlands were predicted to have higher richness losses.

The mean proportional loss of mountain species per cell (i.e., loss in species richness) ranged from 23% under SSP1.26 to 41% under SSP5.85. The number of cells losing more than 50% of their current species ranged between 6% and 53% for SSP1.26 and SSP5.85, respectively. A small number of grid cells (0.9%) lost suitability for all species across all emission scenarios. These sites were concentrated in the Southern African Ranges (0.8% of the total mountain area) and the Central African Highlands (0.1% of the total mountain area). Mostly, they were associated with land use conversion and presented little variation (< 0.05%) across SSPs.

### 3.3 Variable Importance and Land Use Changes

Temperature and soil predictors were the most important explanatory variables (i.e., most frequently having the highest percentage contribution) defining the suitability across all models trained for the present scenario, followed by precipitation (Data S1). Four variables were the most important for 72% of the species compared: soil organic carbon density (179 species), maximum air temperature of the warmest month (147 species), precipitation of the driest month (56 species), and soil organic carbon content in the fine earth fraction (56 species).

Regarding the changes in predominant land use classes projected for African mountains, an overall increase in natural vegetation cover is projected for the sustainability pathway (SSP1.26), while SSP3.70 and SSP5.85 are expected to present declines in most natural land cover categories (except for grasslands). A summary of the changes in land use classes identified for each region is presented as Data S1. Among agricultural categories, rainfed crops (27) and bioenergy crops (29) showed the highest increases under SSP3.70 for the West Africa Mountains and Central African Highlands. Rainfed corn, soybean, and rice croplands also showed moderate increases in the East African Highlands under SSP5.85.

## 4 Discussion

### 4.1 Range and Elevation Shifts in Response to Climate Change

Our results indicate an upslope increase in species richness in upper montane belts across all scenarios, as the mean elevation is projected to increase for most species (75%–85% of the species analyzed, depending on the climate change scenario). In theory, such elevational shifts are facilitated in mountains by the high density of geographically close habitat found across the elevational gradient, offering geographically close equivalents of current temperature belts that are more easily reachable than across latitudinal gradients (Antonelli et al. 2018; Körner and Hiltbrunner 2021). Specifically for Africa, upward displacements of lowland taxa due to climate change have been predicted previously for the Bale Mountains (Kidane et al. 2019), the South African Great Escarpment (Bentley et al. 2019), and the Albertine Rift (Ayebare et al. 2018). Our results are in line with these predictions and provide regional estimates of elevational responses. The mean elevation shift our models project for SSP1.26 (77 m mean increase until 2100) is consistent with the global average of 11 m per decade calculated across taxa worldwide (I.‐C. Chen et al. 2011), but the projection for SSP5.85 (240 m mean increase until 2100) is three times higher than this global average rate. This indicates that compared to near‐present global elevation response rates, African mountain plants may face significant lags in elevation responses that can increase species' vulnerability to regional extinctions. Comparatively, decreasing mean elevation ranges were predicted for 151, 98, and 93 species under SSP1.26, SSP3.70, and SSP5.85, respectively, indicating that some species may increase their occupation of lowlands, most likely undergoing latitudinal shifts instead of elevational ones.

In addition to the increase in mean elevation ranges, contraction in range size was also frequently predicted (299 to 459 species). Such contractions were, in general, higher for species occurring in upper elevations, most likely due to the smaller geographic ranges observed for high‐elevation species. In comparison to global estimates of distribution declines caused by climate change, our models predicted lower changes in suitable areas, ranging from −25% to −55% (without dispersal; contraction only) and +57% to +7% (with dispersal; net change). For a comparison, alpine plant species in the European Alps were projected to lose 44%–50% (under low and high dispersal parameters) of their ranges until 2100. It is important to note, however, that our inclusion of a maximum dispersal scenario may represent a best‐case scenario, which may not be realistic for all species. Some alpine species, like *Lobelia giberroa* (animal dispersal giant perennial herb, Campanulaceae), *Dracaena afromontana* (animal dispersal giant perennial herb, Asparagaceae), and *Veronica abyssinica* (animal dispersal giant perennial herb, Plantaginaceae), did not present substantial range losses in SSP1.26, suggesting that, in lower warming scenarios, some high‐elevation species may be resilient to temperature changes. The higher climatic resilience of some alpine plants has been linked to conservative traits like belowground growth structures and storage organs, and clonal propagation, accumulated during evolutionary adaptation to the harsh environmental conditions found in the upper elevation belts (Körner and Hiltbrunner 2021).

### 4.2 The Influence of Life Forms on Species Responses

Our results show that different life forms varied in their responses, highlighting the importance of considering multiple plant functional types when modeling communities' responses to climate change. Previous studies comparing species in tropical and subtropical mountain ranges showed that elevation shifts are not a uniform response to climate change, with different rates of elevation shifts being observed for different species and life forms (Zu et al. 2023). Our models projecting range contractions across life forms suggest that trees and trees/shrubs may be less vulnerable to climatic changes than herbs, shrubs, and ferns. These predictions are consistent with paleo‐palynological data and vegetation reconstructions available for the Eastern Arc, which suggest that typical Afromontane trees had broader temperature tolerances than other montane elements in past climatic scenarios (Ivory et al. 2016). Taken together, our results suggest that most trees found in Sub‐Saharan African mountains may tolerate the environmental changes projected for the region better than other life forms. Highly diverse responses were projected for herbs and shrub growth forms, suggesting that this category presents a higher variation in their abiotic tolerances and maximum dispersal distances (e.g., types of dispersal and generation times).

Species from families with limited dispersal potential, such as shrubs and herbs with ballistic dispersal in Crassulaceae and Iridaceae and passive wind dispersal in Melastomataceae, Orobanchaceae, and some Asteraceae, had higher mean range losses projected. Meanwhile, families with higher dispersal capacities, such as wind‐adapted herbs in Asteraceae or animal‐dispersed trees in Annonaceae, Lamiaceae, and Sapindaceae, were more frequently associated with increases in potential suitable range. These differences, when combined with range size, geographic drivers, and isolation, may limit the potential for some families and genera to track their suitable climates. Different dispersal adaptations across families can have variable efficiency according to specific environmental conditions, as, for example, some wind‐adapted species from Asteraceae are more dispersal efficient in high elevation habitats (Hintze et al. 2013). Animal‐dispersed species, on the other hand, despite being theoretically capable of reaching longer distances, may be negatively impacted by declines in biotic interactions triggered by climate change.

We found no statistically significant difference between the current distribution area of different life forms (ANOVA *p* > 0.05), indicating that it may not be the reason for the lower proportional distribution losses projected for trees. Trees also did not present higher dispersal capacities than other classes, but still presented some of the higher values of proportional net change across all scenarios compared. Our interpretation is that it is a product of the higher stability of environmental conditions in lower montane forests in comparison to other higher elevation biomes. Nonetheless, differences in vulnerability will also depend on other unmodelled processes, like the slower growth rates and the adaptive potential of trees and their longer reproductive maturity in comparison to other life forms, which may limit range shifts. Also, long‐term adaptive processes may produce different responses across life forms, as the lengths of life cycles between short‐lived (herbs and ferns) and long‐lived (trees) species may allow for faster paces of genetic reshuffling and adaptation in the former (Compagnoni et al. 2021), meaning that trees may be under higher vulnerability considering broader time scales. In addition, particular traits found in trees, such as larger plant height and higher rooting depth, have been argued to provide higher resilience to climate change (Kühn et al. 2021).

### 4.3 Isolation Challenges the Persistence of Taxa in Upper‐Elevation Belts

Our results indicate that species restricted to high‐elevation sites have smaller distribution ranges (Figure 2a), which increases their risk of disappearing regionally under novel climatic conditions (Ohlemüller et al. 2008). Species in upper‐elevation belts were projected to face higher distribution losses, contradicting the argument that they would be more resilient to climate change due to the presence of higher tolerance to harsh environmental conditions (Körner and Hiltbrunner 2021). Even though they may possess enhanced conservative traits compared to some other mountain species, the environmental changes in the lower elevational limits of their distributions may fall beyond their tolerances under higher warming scenarios. This increases the fragmentation of suitable habitats for alpine species, which may be more severe than for other mountain groups, challenging their capacity to compensate for habitat loss via dispersal.

The geographic isolation of mountain peaks and higher proportional distribution loss predicted for high‐elevation species converge to an amplified vulnerability of upper montane and alpine taxa in comparison to low‐elevation taxa. Intermediate‐ and low‐montane species have higher connectivity compared to alpine species, as dispersal may be facilitated by forest bridges (Gizaw et al. 2013) and environmental homogeneity. Meanwhile, the archipelago‐like structure of mountain regions can limit propagule flux for many groups, limiting the potential for range expansions. Also, seed dispersal is being affected by climate and land use change, particularly for plants adapted to animal dispersal. The distribution of birds, mammals, and other fruit dispersers may also shift, and if this is not synchronous in time and space with shifts in plant species, it could affect trophic structures and overall community stability (Blois et al. 2013). Current evidence also supports changes in wind direction and intensity, as well as an increase in the frequency of storms and hurricanes, possibly influencing wind and water dispersed propagules (Comita et al. 2009; Kling and Ackerly 2020).

Land‐use change creates additional challenges to tracking suitable environments by increasing natural landscape conversion into pasture and croplands, reducing connectivity and seed dispersal (Elsen et al. 2020). As mountains include many range‐restricted species and narrow environmental spaces, they are especially vulnerable to land‐use change and its impacts on ecosystem functioning and biodiversity (Adler et al. 2022). Compared to lowlands in Africa, mountains have specific patterns of land‐use change, influenced by topographic complexity and landscape fragmentation (Elsen et al. 2020). African mountains have experienced the highest rates of deforestation (0.5% year^−1^) and the smallest proportional gains (15.4% year^−1^) worldwide (He et al. 2023). Given these circumstances, reducing habitat degradation and increasing restoration and rehabilitation efforts may be necessary not only to ensure the persistence of the most vulnerable species but also to provide conditions for the range shifts to follow the rates of climatic change.

### 4.4 Regional Patterns of Biodiversity Loss

The impacts of climate change on upslope range shifts are projected to be more pronounced for mountain species near the tropics (Colwell et al. 2008). While our models predict species richness losses with a slight peak close to 20° S and to 20° N, no general latitudinal trend across species was detected. Latitudinal changes were observed to vary across scenarios, with higher emission scenarios linked to more frequent poleward shifts, particularly in the Northern hemisphere (49% of the modelled species increasing their maximum latitudes under SSP585, vs. 35% under SSP370 and 39% under SSP126). Regarding individual species responses, the geographic structure of the mountains in Africa appears to have an important influence on driving this response.

Poleward shifts are limited in most African mountains as they are highly fragmented and have few linear components along latitudinal gradients. Some examples of northward migration were projected for species concentrated in the South (Drakensberg and the Cape Floristic region). Within this region, most projected suitability gains were northeasterly, where the closest neighboring mountain ranges are located (the Eastern Highlands between Mozambique and Zimbabwe). This pattern is due to the absence of landmasses southwards, given the position of these mountain ranges in the southernmost part of the continent. The combination of this geographic limitation with limited dispersal capacities hinders latitudinal shifts for many Cape species, such as *Chrysocoma ciliata* (wind dispersal shrub, Asteraceae), *Dimorphotheca cuneata* (wind dispersal shrub, Asteraceae), and most *Crassula* species (ballistic dispersal shrubs and herbs, Crassulaceae). Meanwhile, species with similar distribution ranges but higher dispersal capacity, such as *Buddleja saligna* and *B. salviifolia* (animal dispersal shrubs or trees, Scrophulariaceae), *Cussonia spicata* (animal dispersal tree, Araliaceae), *Burchellia bubalina* (animal dispersal tree, Rubiaceae), and *Tetradenia riparia* (animal dispersal shrub or tree, Lamiaceae) may be capable of northward latitudinal shifts, as projected by our models. Comparatively, mountain regions with higher connectivity across latitudinal gradients, such as the Rift Mountains (e.g., Albertine Rift, Southern Rift), showed more frequent examples of latitudinal shifts for species, particularly the ones with higher dispersal capacities. Some examples are *Acalypha psilostachya* (animal dispersal herb or shrub, Euphorbiaceae), *Allophylus abyssinicus* (animal dispersal tree, Sapindaceae), *Neoboutonia macrocalyx* (animal dispersal tree, Euphorbiaceae), and *Rutidea orientalis* (animal dispersal shrub, Rubiaceae).

These results, despite not representing a single shared pattern of latitudinal response, demonstrate how distribution shifts will not only be constrained by dispersal but also by geographic structure and distance between mountain ranges. For most mountain species, dispersal is already limited by major topographic intervals, and that will be augmented by a widening sea of warmer temperatures and probably aridity/extreme weather events (e.g., heatwaves and ENSO‐linked events) within these intervals (Adler et al. 2022). This will have a major role in defining whether species will have suitable habitats available across an accessible dispersal distance, particularly for isolated mountain ranges like the Cape Floristic Region and the Malagasy Mountains.

In western Africa, predicted losses in species distributions were mild and an increase in species richness was projected for all scenarios. This pattern aligns with the scenario of drought‐sensitive species migrating towards mountains, previously suggested by regional comparisons of climatic models, due to the projected increase in orographic precipitation for the Guinean Highlands and the Jos Plateau in the next decades (Sylla et al. 2016). On the other hand, the human population, agriculture, and mining in West Africa are projected to increase (Adepoju et al. 2019), possibly imposing even higher threats to the persistence of the regional biodiversity than climate change.

In the Central African Highlands, losses were projected along the eastern slopes of the Cameroon Highlands, especially under the moderate and high emission scenarios (SSP3.70 and SSP5.85). This region includes some of the highest mountains in western Africa, such as Mount Cameroon (4095 m), Mount Oku (3011 m) and the Bamboutos Mountains (2740 m), and some of the highest concentrations of vascular plant diversity on the continent (Onana and Cheek 2011). The vegetation comprises a mosaic of Afromontane forests, lowland tropical forests, and grasslands. Climate change projections indicate that northern Cameroon, where most of the declining cells were identified, is expected to face higher increases in temperature and more frequent temperature extremes than the south (Gloy et al. 2023), where we identified increases in species richness. During the Holocene climatic fluctuations, pollen records available for Cameroon show that the upper montane forests were relatively unstable across warming‐cooling cycles compared to their lower limits (Lézine et al. 2019), suggesting that higher elevation sites might be more sensitive to future warming, as shown by our predicted changes in species richness. Considering land use changes, the most relevant threats to plants in the Central African Highlands are overgrazing and agriculture (Onana and Cheek 2011), which are projected to show the highest proportional increase under SSP3.70 and SSP5.85 among compared regions (see Data S1).

For the Southern African Ranges, projected losses were concentrated in Angola, in the Bié Escarpment, and, under moderate and high emission scenarios, in different portions of the Great Escarpment across South Africa, Mozambique, and Zimbabwe. Angola has been highlighted as a notorious gap in African mountain biodiversity research, while the other sites have been more thoroughly surveyed recently (Clark et al. 2011). The southern part of the Bié Escarpment, where the levels of species richness decline were particularly acute, exhibited a transition between Afromontane forests and grassland mosaic, to arid shrubland vegetation in the south (Ayebare et al. 2018). In the south and west, high‐richness areas like the Bvumba and Chimanimani Massifs and the Limpopo‐Mpumalanga‐Eswatini Escarpment, and some sites across the eastern part of the Cape showed declines for moderate and high warming scenarios. These declines might be associated with the vulnerability of these regions to desertification (Huang et al. 2020).

In addition to limitations in species' dispersal capacity, the low availability of environmental spaces in landscapes of low topographic heterogeneity may also limit potential range shifts, as there are no upward elevation tracking options for plateaus like the Highveld in South Africa and the summit of the Maloti‐Drakensberg (old erosional land surfaces with limited relief). Surprisingly, species richness was projected to increase for upper regions of the Drakensberg, most likely due to upslope shifts of warm‐adapted lowland taxa—like C_4_ grasses (De Deus Vidal et al. 2021). It is noteworthy, though, that this result may be biased for under‐sampled regions like the Drakensberg. The incomplete listing of the native ranges of some species can artificially enhance the positive changes in richness projected by our models, as we used native ranges as a mask for the present models. Therefore, many species reported to increase their ranges into the region may already be present in those sites, even though they have not been recorded yet.

The East African Highlands encompass the Ethiopian Highlands and the Rift Valley, including Mount Kilimanjaro, Africa's highest mountain peak (5895 m). This region also includes the continent's largest Afroalpine continuous area above 3000 m, found in the Bale Mountains (Kidane et al. 2019). For this region, our models indicate consistent declines in richness for the central and southern Rift Valley across all scenarios compared. For the Ethiopian Highlands, declines were projected for SSP3.70 and SSP5.85, while increasing richness was predicted under SSP1.26. Besides climate change, overgrazing has been reported to be the most relevant threat to the conservation of natural vegetation (Kidane et al. 2019). In addition, even though the agricultural area is projected to remain relatively stable across SSPs, the decrease in indigenous coffee productivity caused by climate change may accelerate deforestation and land‐use changes in Ethiopian mountains (Davis et al. 2012).

While Madagascar's central ranges exhibited high projected losses (Figure 4), in contrast, its overall species richness was projected to increase due to dispersal (Figure 3). The influx of continental propagules in Madagascar is, however, less likely in comparison with other regions included in our analysis because the isolation by the Indian Ocean imposes a stronger filter to dispersal than the lowlands in the African continent (Antonelli et al. 2022). Although our models show that environmental conditions may increase suitability within Madagascar for some species shared with continental Africa, their dispersal may be limited. Climate change projections for the island indicate increasing drought frequency and wildfires (Ingram and Dawson 2005), potentially affecting species expansion and resilience. Additionally, the introduction of novel (and, occasionally, invasive) species by humans has been linked to multiple extinction events along the Quaternary (Ralimanana et al. 2022), indicating that future increases in suitability may enhance the vulnerability of the island biota to the arrival of invasive species.

### 4.5 Uncertainties and Limitations

Although niche modelling provides a useful tool to assess the broad‐scale impacts of climate change on species ranges, its implementation poses some methodological limitations, especially for Africa, where historical climatic data for high‐elevation environments is sparse (Zurell et al. 2023). The uncertainty associated with fine‐scale climatic projections limits the extent and the resolution to which range shifts can be quantified and mapped under complex topographic landscapes such as some African mountains. While the coarse spatial scale of our models (0.05 degrees) can be informative for the regional level, they should be carefully interpreted for small‐scale inferences. In mountains, microclimatic conditions can play an important role in creating environmental variability and may influence range shifts by enabling the persistence of species even in the face of regional climatic changes. Therefore, our results should be interpreted carefully considering the broad scope of our study, and species with narrow climatic niches and limited geographic distributions (e.g., endemics to single mountain regions) need further investigations to robustly estimate their distribution losses under warming temperatures.

In addition, climate‐based distribution models do not account for important biotic factors potentially affecting species distribution, such as competition and predation, nor all the biological processes behind the responses to climate change, which might include phenotypic plasticity or local genetic adaptation (Zurell et al. 2023). One such example is the impact of the increasing establishment of invasive species in mountain environments, which may synergistically accelerate the rates of biodiversity loss in mountain regions by excluding native colonization. Another limitation is the so‐called rare species paradox, where species with few records cannot be reliably modeled. Species with distributions restricted to high‐elevation belts are therefore under‐represented in our findings, especially for areas with poor sampling coverage and low availability of digitized records. To improve our understanding of the ongoing responses to climate change, we advocate increasing efforts to digitize collections and to improve the sampling in mountain regions, not only in Africa but throughout the tropics.

While our estimates of dispersal provide a more refined proxy for the potential range expansion of individual species, our models simplify some important ecological and morphological traits that may influence the actual dispersal capacity of species. The classification of dispersal syndromes and growth forms, in practice, is more complex than the categories adopted in the models. The variability in individual sizes, seed production, spatial distribution, and even ecological interactions can produce a wide variation in the dispersal distances considered for each species. The maximum dispersal distances adopted for our model projections will most likely not be fully achieved in reality, as they were calculated to provide a maximum range estimate and not probabilities of distribution of propagule movements, which would be much more complex to simulate.

### 4.6 Implications for Climate Change Adaptation and Biodiversity Conservation

Among the 607 species modelled in our study, only 189 (ca. 31%) have been assessed on the IUCN Red List. Most of the assessed species are considered Least Concern (184). Only three are Near Threatened, namely *Ocotea racemosa* (animal dispersal tree, Lauraceae), *Psychotria zombamontana* (animal dispersal shrub or tree, Rubiaceae), and *Vepris suaveolens* (animal dispersal tree, Rutaceae). Only two species are listed as threatened under the Vulnerable category, namely *Cordia platythyrsa* (animal dispersal tree, Boraginaceae) and *Prunus africana* (animal dispersal tree, Rosaceae). A comparison with our results indicates that up to 132 Least Concern and 325 non‐evaluated species may experience a contraction in distribution under all climate change scenarios. More than one‐third of all African plant species are estimated to be currently threatened following the IUCN Red List criteria (Stévart et al. 2019). While the IUCN Red List can incorporate climate change‐associated risks, it currently does not include many assessments that have identified and projected distributions into future climates, due to the high complexity and uncertainties involved in identifying and making these projections (Zurell et al. 2023). Therefore, identifying potentially vulnerable mountain species in African mountains can take advantage of information such as elevation ranges, life forms, dispersal limitations, and range sizes, which are easier to calculate and can be applied in combination with an expert‐compiled Red List assessment to improve the conservation of species with higher climatic vulnerability.

## 5 Conclusion

We demonstrate that the persistence of alpine and upper montane elements of the mountain flora in Africa will depend on elevational shifts and range expansions that can only occur if the connectivity of habitats and the functioning of biotic interactions are safeguarded. Our results show that upper‐elevation species may be more vulnerable to net losses, which is even more concerning given the isolation level of upper montane habitats. Different climate and land use change scenarios will determine the pace at which these responses may occur. Meanwhile, current global rates of elevation shifts suggest that under SSP3.70 and SSP5.85, many African species will not be able to compensate for range losses. In cases of imminent threats that cannot be reversed, human‐assisted migrations and *ex‐situ* conservation (such as through seed banking) should be considered.

## Author Contributions

**João de Deus Vidal Junior:** conceptualization, data curation, formal analysis, funding acquisition, investigation, methodology, project administration, resources, software, validation, visualization, writing – original draft, writing – review and editing. **Alexandre Antonelli:** conceptualization, methodology, supervision, validation, writing – original draft, writing – review and editing. **Clinton Carbutt:** data curation, investigation, supervision, validation, writing – review and editing. **Vincent Ralph Clark:** data curation, investigation, supervision, validation, writing – review and editing. **Tobias Fremout:** formal analysis, investigation, methodology, writing – review and editing. **Christopher Chapano:** data curation, writing – review and editing. **Inês Chelene:** data curation, investigation. **David Chuba:** data curation, writing – review and editing. **Tadesse Woldemariam Gole:** data curation, writing – review and editing. **Clayton Langa:** data curation, writing – review and editing. **Benoit Loeuille:** data curation, writing – review and editing. **Ermias Lulekal Molla:** conceptualization, data curation, writing – original draft, writing – review and editing. **Timothy Richard Pearce:** data curation, investigation, resources, writing – review and editing. **Andrew J. Plumptre:** data curation, validation, writing – original draft, writing – review and editing. **Feyera Senbeta:** data curation, writing – review and editing. **Carolina Tovar:** conceptualization, data curation, formal analysis, methodology, project administration, supervision, writing – original draft, writing – review and editing. **Joseph Douglas Mandla White:** conceptualization, data curation, methodology, project administration, writing – original draft, writing – review and editing. **Christine Brigitte Schmitt:** conceptualization, data curation, formal analysis, funding acquisition, investigation, methodology, project administration, resources, supervision, validation, visualization, writing – original draft, writing – review and editing.

## Conflicts of Interest

The authors declare no conflicts of interest.

## Supporting information


**Data S1:** gcb70492‐sup‐0001‐DataS1.zip.

## Data Availability

All scripts, data, model outputs, individual species maps, and projections are available both as Supplementary Files and on Zenodo [https://doi.org/10.5281/zenodo.16948754].
